# Political Imagination, Otherness and the European Crisis

**DOI:** 10.5964/ejop.v11i4.1085

**Published:** 2015-11-27

**Authors:** Vlad Petre Glăveanu, Constance de Saint Laurent

**Affiliations:** a Aalborg University, Aalborg, Denmark; b University of Neuchatel, Neuchatel, Switzerland

“Germany caused this problem. And they're eventually going to give every single migrant German passports - they will all be flooding the UK within 5 years. And they'll all be voting for the Labour party. And we'll be trapped in the EU by non-indigenous British voters for eternity”

(Lewis, commenting on the article ‘German interior minister calls for limits on migrants to EU’ published by Yahoo News, 9 September 2015)

All those following media reports in Europe over the past seven years will be forgiven for thinking that the continent is in perpetual crisis^i^. Whether a social, economic or political one, the same formula seems to apply: collective frustration over the current state of affairs; sense-making about the situation; finding culprits, preferably from outside one’s group or nation; protesting, more or less vocally, and moving on to the next crisis. At each stage of the ‘process’ a plethora of journalists, economists, sociologists and political scientists readily offer their views, lament or denounce the status quo, compare what happens here and now with what happened then and there, and generally accompany social movements as they unfold. What can psychologists contribute to these debates?

In this editorial we argue that a psychological perspective is not only needed but fundamental for understanding current events. Lewis’s comment, included as a suggestive motto, captures the essence of what psychology, we believe, can contribute with. The short quote is overloaded with evidence of various psychological processes applied to the migration crises and its effects on the UK (presumably Lewis being a resident of this country). There is evaluation (‘Germany caused this problem’), anticipation (‘they’re eventually going to give every single migrant German passports’), causal thinking (‘they will all be flooding the UK within 5 years’), generalisation (‘they’ll all be voting for the Labour party’), and in/out-group dynamics (‘we'll be trapped in the EU by non-indigenous British voters for eternity’). All these mobilised for political aims, since an implicit argument is being built here that Germany should not receive migrants, migrants should not get German passports and move to the UK, the fate of the country should not be determined by ‘non-indigenous’ voters and (perhaps) the Labour party should stop wanting Britain trapped inside the EU. Still, rarely do psychologists write about political implications; they are more comfortable instead focusing on the nature and mechanisms involved in making evaluations, generalisations and in thinking in general, particularly its biases and heuristics (see Kahneman, 2011). While this is a worthwhile enterprise in its own right, we believe it to be falling short of the full potential this discipline has to study, address, and contribute to social change.

As follows, we will introduce imagination as a key psychological concept for theorising social change and define the notion of political imagination. We briefly discuss different elements included in this definition and, in particular, outline the relation between politics and imagination. In the end, we offer some reflections on both the ‘dark’ and ‘bright’ sides of political imagination as it participates in the construction of society in the spirit of nationalism and exclusion or, on the contrary, mobilises people to fight for just and democratic political processes. By understanding the dynamic of political imagination, psychologists would have a lot to contribute to current social debates in Europe.

## Imagination, Otherness and Society

We propose to expand the discussion of psychological processes involved in social change from biases in cognition to the work of human imagination. Bringing in the notion of imagination might seem odd considering its relegation today to the fields of philosophy, aesthetics, and the arts (Cornejo, 2015). And yet, everything about Lewis’s quote points to the use of this faculty. There are imagined events, imagined consequences and, on the whole, an imagined future constructed in just a few lines. Arguably, Lewis is not experiencing, at the moment of writing, any of the anticipated consequences of migration; in fact, he might not even have met, in person, any of the migrants arriving to Europe in the wake of the Syrian conflict, little less asked them about their plans in 5 year’s time. However, in his narration, the future of living with (and under) these other people is made ‘real’ and this infuses Lewis’s imagination, arguably, with all sorts of images and scenarios about what it will mean to live in a country full of migrants, run by the Labour party and trapped in the European Union… These future scenarios are thus built on a fundamental and deeply consequential form of imagination – the imagination of otherness.

It is important at this point to clarify our stand in one of the longest debates concerning human imagination – that of its relation to reality. Drawing on sociocultural psychological scholarship (Vygotsky, 2004), we consider imagination as a process deeply connected to how we experience the world at every moment. In fact, imagination participates in perception itself (Pelaprat & Cole, 2011), helping us construct images of what ‘is’, in addition to what is ‘not’ or ‘not yet’. In building reality, imagination draws on existing cultural resources, as well as past experience, in ways that enrich our here and now (bringing in the general, the possible, the impossible) and open it to new temporal dimensions (such as the future, the past we have not experienced, etc.) (see Zittoun & Gillespie, 2015). Considering this complexity, the study of imagination in psychology and connected disciplines is rightfully multi-faceted. It ranges from understanding how children engage in pretend play episodes (Harris, 2000) up to how we creatively construct, in time, a personal life trajectory (Zittoun & de Saint-Laurent, 2014). For the purposes of our discussion here, however, we are interested in a particular form of imagination, one that concerns collective rather than individual life. This is not an imagination by society, performed by a mysterious ‘group mind’, but one *of* society, developed by people as they engaged in collective living. As such, it is an imagination at once individual and social, symbolic (grounded in representations) and material (grounded in physical and institutional arrangements); it is also, ultimately, a political form of imagination, even when those who imagine do not directly participate in the institutionalised arena of politics.

## Political Imagination

What do we define as political imagination? In a broad sense, this concept is meant to designate all those *imaginative processes by which collective life is symbolically experienced and this experience mobilised in view of achieving political aims*. Almost all the concepts above require some qualification.

### Imaginative Processes

What do we mean by imaginative processes? They refer to those psychological mechanisms that allow us to bring into the present, the here and now, other experiences (Glăveanu, Karwowski, Jankowska, & de Saint Laurent, forthcoming). These experiences can be spatially or temporally distant, but also impossible or improbable. Through this move, we transform our understanding of the present and how we act towards it.

This kind of approach resonates with sociocultural definitions of imagination put forward in recent years (Pelaprat & Cole, 2011; Zittoun & Cerchia, 2013), definitions that escape the narrow understanding of this process in terms of imagery alone. It also invites us to reflect on who engages in such processes. By considering them as primarily psychological, we propose to study political imagination at the level of the individual; however, this is always an individual that exists within a collective or social context. In this sense, the products of political imagination are never, themselves, individual in nature.

What scaffolds, then, political imagination? We postulate that a wide range of social and educational experiences offer individuals the resources to imagine, in line with Vygotsky’s emphasis on the cultural roots of imagination (see Vygotsky, 1994, 2004). In other words, the collective life this form of imagination is directed towards also offers people the concrete means to imagine.

### Collective Life

What do we understand by collective? Broadly speaking, ‘collective life’ designates all aspects of life that are the product of people acting as part of a group. Benedict Anderson (1983) made an important contribution to this discussion in his book on imagined communities. A popular idea in sociology and politics, his argument is that big social groups, particularly nations, are always imagined since “even the smallest nation will never know most of their fellow-members, meet them, or even hear of them, yet in the minds of each lives the image of their communion” (p. 15). Thus, experiencing the social world necessarily involves, at least in part, imagination. However, we would like to emphasise the fact that imagination should never be reduced to our relation to an absent other. On the contrary, political imagination is political precisely because it informs if we accept or not, understand or not, engage or not with very concrete others.

### Symbolic Experience

Then why talk first and foremost about symbolic experience? Does political imagination not have a material, embodied side as well? It certainly does. But the notion of symbolic highlights the construction of imaginative experiences with the use of images, language, social representations, etc. This doesn’t mean that such symbolic means don’t have both concrete referents in the world or take materialised forms (in writing, drawing, talking, acting, and so on), but that their efficiency lies in their ability to connect what is here and what is not in a specific situation, to be a symbol of something else.

### Political Aims

Last but not least, the key notion of ‘political aims’ needs to be clarified. As briefly mentioned before, we are not concerned here only with those manifestations of imagination within the socially defined and institutionally enforced area of the political. For us, political aims concern others and how one ‘deals’ with otherness (and, through this, with oneself in relation to this otherness). What political aims are pursued in relation to others? To assimilate, exclude, control, dominate, emancipate, empower… and the list goes on. We therefore take ‘political’ in its double meaning, referring both to general matters of public life and to the ‘policing’ of social groups.

## Imagination and Politics

In his paper, ‘Politics at its best: Reasons that move the imagination’, Ferrara (2011) offered not only a good, simple definition of politics, but also explicitly and convincingly related politics with imagination. In his words:

“First, no human being exists who does not act and whose action does not fit, albeit only in a merely mental sense, within a larger human collectivity. Second, no action can be envisioned without reference to some notion of ends and means. Third, no preestablished harmony exists between all the ends pursued by human beings and the social unions within which they live. Hence the need for politics: politics sinks its roots in the unavoidable necessity of coordinating the ends of one’s own action with those underlying other people’s actions when we live in a shared world” (p. 39).

Imagination is, for him, an essential part of any political process:

“Only a human form of association to which unlimited resources were available and which could equally satisfy all the ends striven after by all of its members could dispense with politics. The important role of imagination becomes manifest here: by enabling us to project an image of the world, the imagination allows us to perceive certain ends as deserving more or less priority over others and, more particularly, to envisage new ends” (p. 40).

Importantly for Ferrara, and for our concept of political imagination, not all prioritisation of ends falls within the domain of politics. Choosing ends that concern personal life, for instance, doesn’t require political deliberation. In other words, they belong to the private rather than the public domain. For Ferrara (2011, p. 41), “only that deliberation on the priority of ends is political which – either on account of the nature of the controversy, or on account of the large number of people entitled to participate, or on the account of the mode of deliberation, or on account of all or some combination of these elements – produces outcomes which are binding for everybody” (p. 41). These comments help us define the realm of political imagination; however, they tell us little about its processes.

## The Processes of Political Imagination

What are, then, the mechanisms involved in political imagination? The first step, we assume, concerns building representations of others, their goals and intentions within collective life. The second, uses these representations to influence (limit or enhance) the possibility of others achieving their (imagined) aims. These two ‘phases’ are of course cyclical and interdependent since constructing otherness defines how one acts on this construction which, in turn, impacts the representation, and so on. What is important to note here is that, while a lot of discussion of imagination (and, for this matter, of political imagination; see Bottici & Challand, 2011), focused mainly on the construction of images, we are not referring here exclusively to images but rather their coordination within scenes or scenarios that constitute experience (Glăveanu et al., forthcoming). We draw in this regard on the philosophical perspective of Vendler (1984) who pointed out the fact that we never imagine separate, fully formed images but scenes.

Let us return to Lewis’s remarks concerning immigrants. While an implicit image of the immigrant is being built here (as someone who is dangerous, opportunistic, oppresses the local population, votes for Labour and supports the EU, etc.), it is in fact imagined actions that take centre stage. A similar phenomenon was studied by one of the authors in relation to how Romanians imagined EU citizens and how they thought EU citizens saw them in the eve of Romania’s accession to the European Union (Glăveanu, 2007). The auto and hetero-stereotypes revealed by this study were, in turn, articulated within relational scenarios, including in a metaphorical manner (in which, for example, Romania plays, for most respondents from Bucharest, the part of Cinderella, and the EU that of the prince or, for some, the wicked stepmother).

The empirical study of political imagination as a concept has a short history but a long past. Indeed, without always using this notion – or even the notion of imagination itself – extensive research has been conducted on issues related to what we define here as political imagination in the social sciences as well as in community, political, critical, decolonial, and liberation psychology. Many such studies were made available to the general public. To take only one example, Daniel Francis (1992) first authored, more than two decades ago, a fascinating book on the ‘Imaginary Indian’ (republished in 2011). In this book, he set out to “understand where the Imaginary Indian came from, how Indian imagery has affected public policy in Canada and how it has shaped, and continues to shape, the myths non-Natives tell themselves about being Canadians” (p. 22). In structuring the discussion, Francis organised his presentation along the following sections: 1) taking the image; 2) presenting the image; 3) appropriating the image; and 4) implementing the image. His approach is thus certainly very close to the two step mechanisms proposed above. Other analyses of political imagination, closer to the migration debate in Europe, are offered in recent chapters on how race is perceived in the West (Lentin, 2011) and the struggle for people’s imagination of Islam (Challand, 2011). In the latter, a thorough presentation is included on how Islam “gradually emerged as one of the most significant Others of our epoch and has often served as negative counterpoint to the representations of modernity, democracy and western identity” (p. 142).

## The ‘Dark’ and ‘Bright’ Sides of Political Imagination

Faced with these illustrations, together with the grim picture of a Europe divided on the issue of immigration, particularly from Muslim countries, it is legitimate to ask whether political imagination is not, in the end, the dark side of imagination. This is all the more striking when contrasted with recent literature arguing for the crucial role imagination plays for the development of the individual as well as the development of culture. Zittoun and Gillespie’s (2015) scholarly volume on this topic covers a wide range of examples in this regard, from imagination in the life course to imagination and social change (the latter through the power of utopias and the emergence of ‘communities of imagination’). Observing the many crises of Europe in the past decade compels us to reflect, however, also on situations in which imagination can lead to exclusion, discrimination and the maintenance of the status quo. Moreover, it is not only the presence but ‘absence’ of political imagination that can carry terrible consequences (for a similar argument see Bottici & Challand, 2011). How else can understand the responses to the Greek financial crises that presented the country no other alternatives than that of welfare cuts and neo-liberal reforms? Or the situation whereby, in Europe, the agenda of incessant ‘growth’ has no rival or, at least, no rival strong enough to win over people’s imagination? In this macro context, the relation between the lack and ‘dark’ side of collective imagination is evident, leading to imaginative scenarios of the following kind found, for instance, on the website of the French extreme right party, the National Front:

Migrants come to our countries to take advantage of social welfare

But our countries are facing an economic crisis

So we should close our boarders to protect ourselves

But we cannot because of the Schengen agreements and the quotas imposed by the EU

Therefore we need to leave the EU

Here, the premise is the imagination of an Other, and the kind of actions his imagined aims and values will lead to, i.e., taking advantage of the European social welfare and potentially threatening its security. This kind of political imagination unfolds before us in the media, in political discourses and in everyday conversations, and it leads to specific actions in the present. Here, it encourages leaving the European Union, but also, implicitly, racist and discriminatory responses to the migration crisis.

But is the picture so grim? Reducing it to the development of nationalist and racist discourses – for as important as they are to study and understand – doesn’t do full justice to the power of a psychological function that animated people, through the centuries, towards developing more fair, democratic and equalitarian societies. Should we not acknowledge its role in sparking the revolutions of the Arab spring? Or animating millions of people to rally in France in defence of ideals such as freedom of speech? Or that, today, it brings young Romanians to the streets in search of alternatives for a political regime based on nepotism and corruption? Political imagination is at work in all of these instances, allowing us to imagine better futures and giving us a sense that there is a collective will to make the world a better place. And it doesn’t necessarily always lead to outright social mobilisation. Its processes are more pervasive and embedded within the everyday lives of people who struggle against inequality and oppression. Jovchelovitch (2015), for example, illustrated in this regard the ways in which imagination is at the core of social resistance and transformation in Rio de Janeiro’s favelas. Without imagination, particularly political imagination, human agency would be impossible since the assertion of one’s agency is, itself, a political project. Neither would our experience of sociality and communal living. The bonds that keep us together are maintained by imagination just as much as they are by direct and mediated contact.

## Concluding Thoughts

It is this last observation that we find particularly important to conclude with. That is, the processes of political imagination – constructing otherness and acting based on this construction – are not meant for good or evil. They are part and parcel of our social life and, just as they support imaginations of separation and oppression, they can and do foster social activism, underpinning all those acts by which, for instance, citizens organised themselves to welcome migrants all along their route through southern and central Europe. It was an imagination of people working together to help and alleviate suffering that arguably saw so many bring food, drive migrants across borders and even open their homes to them and their families. A comprehensive theory of political imagination needs to account for both sides and, in doing so, recognise the strong ethical dimension situated at the very core of this concept. If political imagination deals with otherness then it cannot but ultimately deal with values and the moral dilemmas that mark Self – Other relations. Psychologists have a lot to say about this topic and our hope is to see more of our colleagues contribute to it. In the end, any crisis, including the European one, is also an opportunity.
